# Supplementary material for: Crystal structure and functional implications of cyclic di-pyrimidine-synthesizing cGAS/DncV-like nucleotidyltransferases
Source: Nat Commun. 2023 Aug 21;14:5078. doi: 10.1038/s41467-023-40787-9 (PMC10442399; doi:10.1038/s41467-023-40787-9)
Supplement: Supplementary file 1 — Supplementary Information [file 41467_2023_40787_MOESM1_ESM.pdf]

## Supplementary Figures

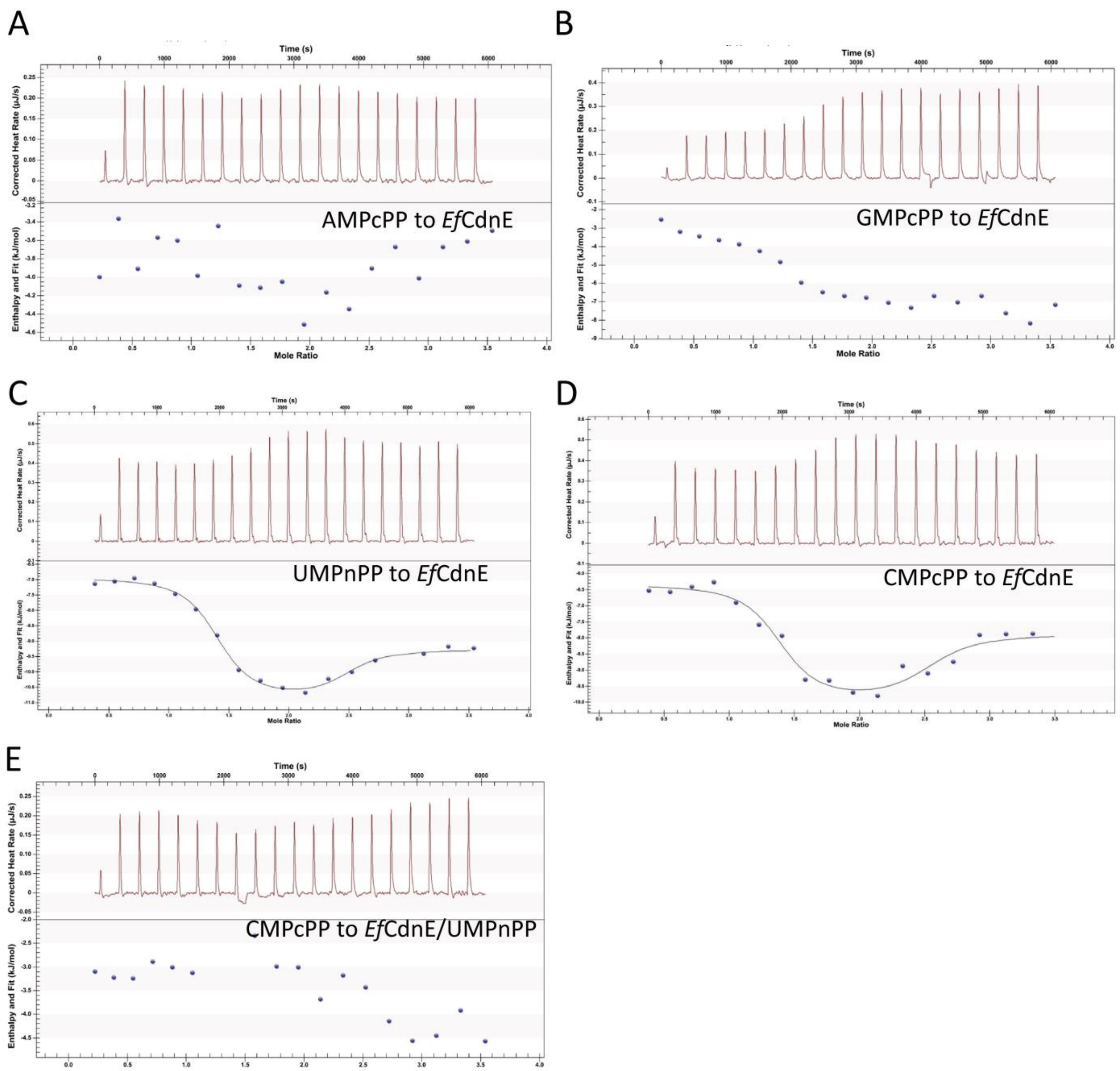


**Supplementary Figure 1. ITC analysis of the nucleotide binding to *Ef*CdnE.**

(A-B) No binding was observed when *Ef*CdnE was titrated with AMPcPP (A) or GMPcPP (B). (C) The titration profile of 500 µM UMPnPP to 50 µM *Ef*CdnE showed two different binding sites with dissociation constants of 1.47 x $10^{-8}$ M and 9.8 x $10^{-7}$ M. (D) The titration profile of 500 µM CMPcPP to 50 µM *Ef*CdnE showed two different binding sites with dissociation constants of 1.87 x $10^{-8}$ M and 1.27 x $10^{-6}$ M. (E) CMPcPP showed no binding to *Ef*CdnE pre-incubated with UMPnPP.

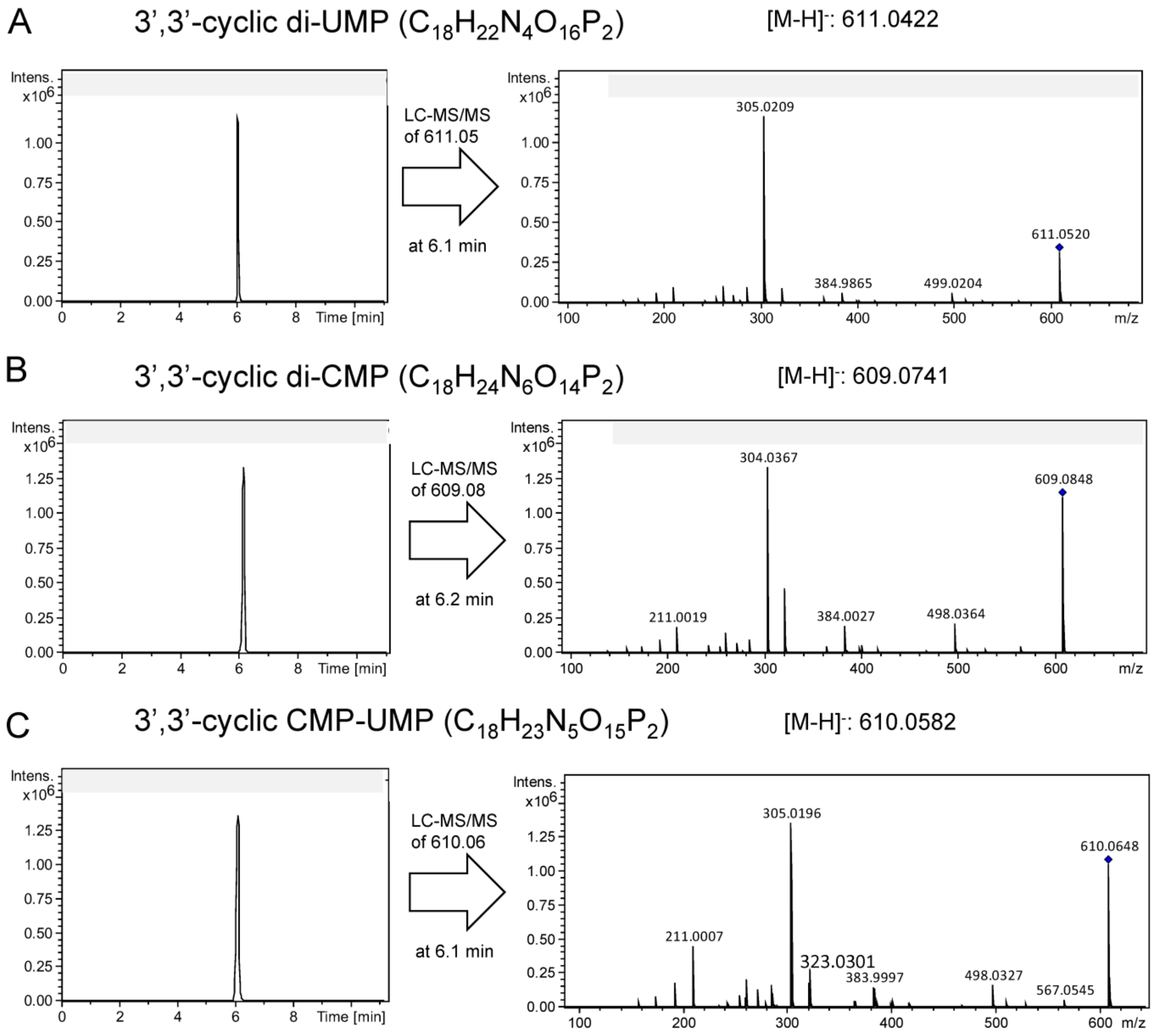


**Supplementary Figure 2. LC-MS/MS analysis of the chemical standards, cUU, cCC and cUC.**
(A) LC-MS/MS result of cyclic di-UMP (theoretical $[M\text{-}H]^-$: m/z 611.0422), which is eluted at 6.1 min and has fragmented ions of m/z 305.0209, m/z 384.9865, and m/z 499.0204. (B) LC-MS/MS result of cyclic di-CMP (theoretical $[M\text{-}H]^-$: m/z 609.0741), which is eluted at 6.2 min and has fragmented ions of m/z 211.0019, m/z 304.0367, m/z 384.0027 and m/z 498.0364. (C) LC-MS/MS result of cyclic CMP-UMP (theoretical $[M\text{-}H]^-$: m/z 610.0582), which is eluted at 6.1 min and has fragmented ions of m/z 211.0007, m/z 305.0196, m/z 323.0301, m/z 383.9997, m/z 498.0327 and m/z 567.0545.

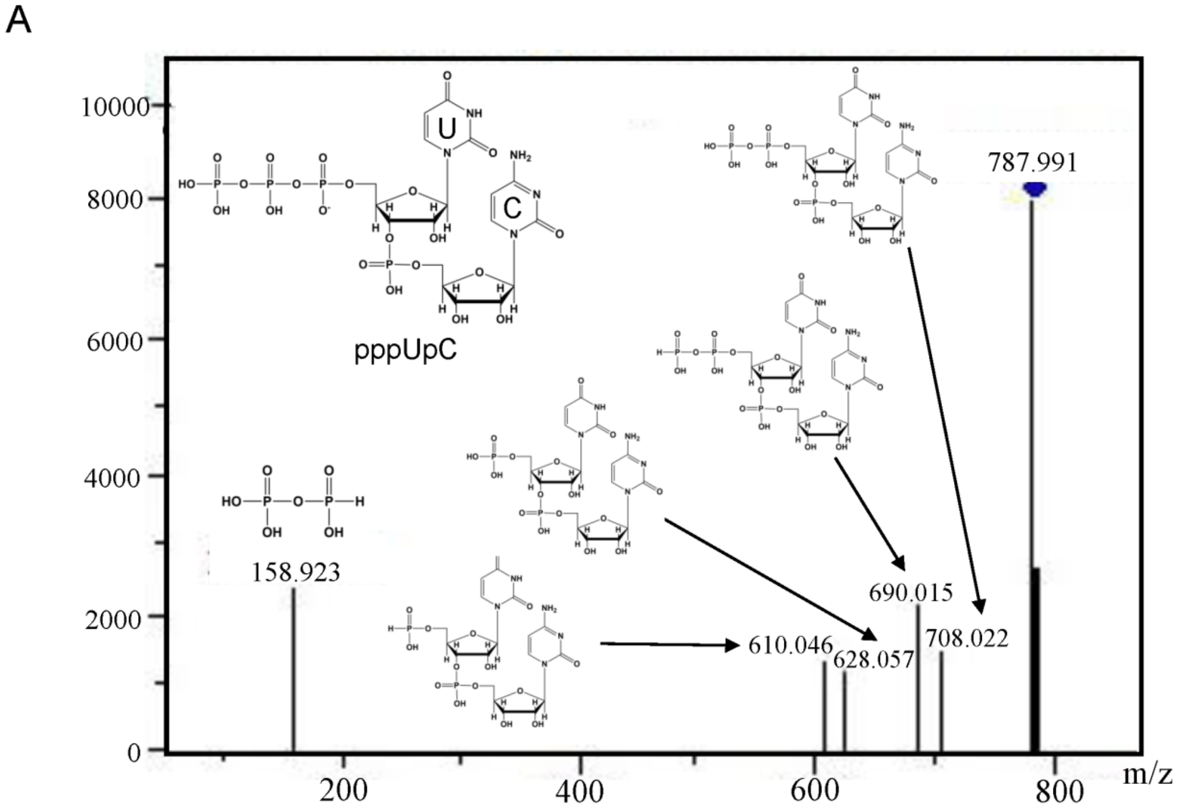


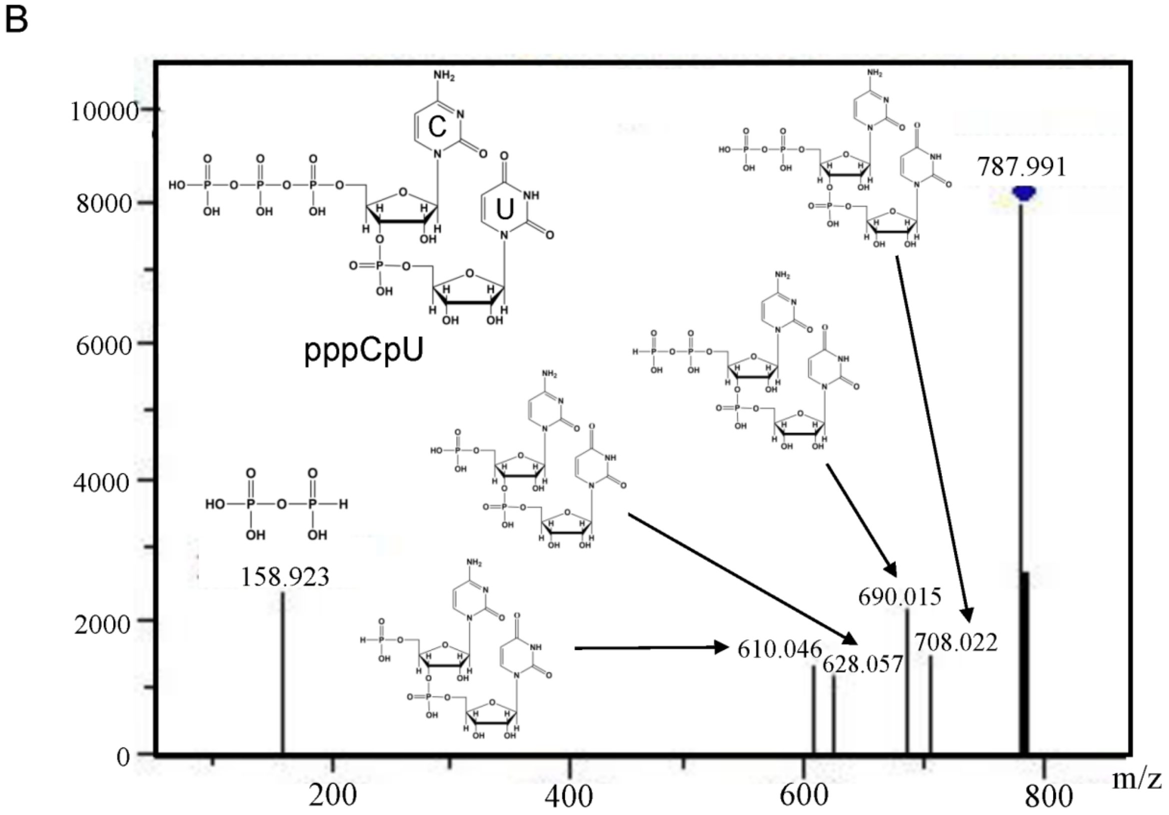


**Supplementary Figure 3. LC-MS/MS analysis of the intermediates generated by *Ef*CdnE using CTP and UTP as substrates.**

The EIC of a fragment ion (m/z 788.00), corresponding to (A) pppUpC or (B) pppCpU. The chemical structures corresponding to each fragment ion derived from (A) pppUpC or (B) pppCpU are shown.

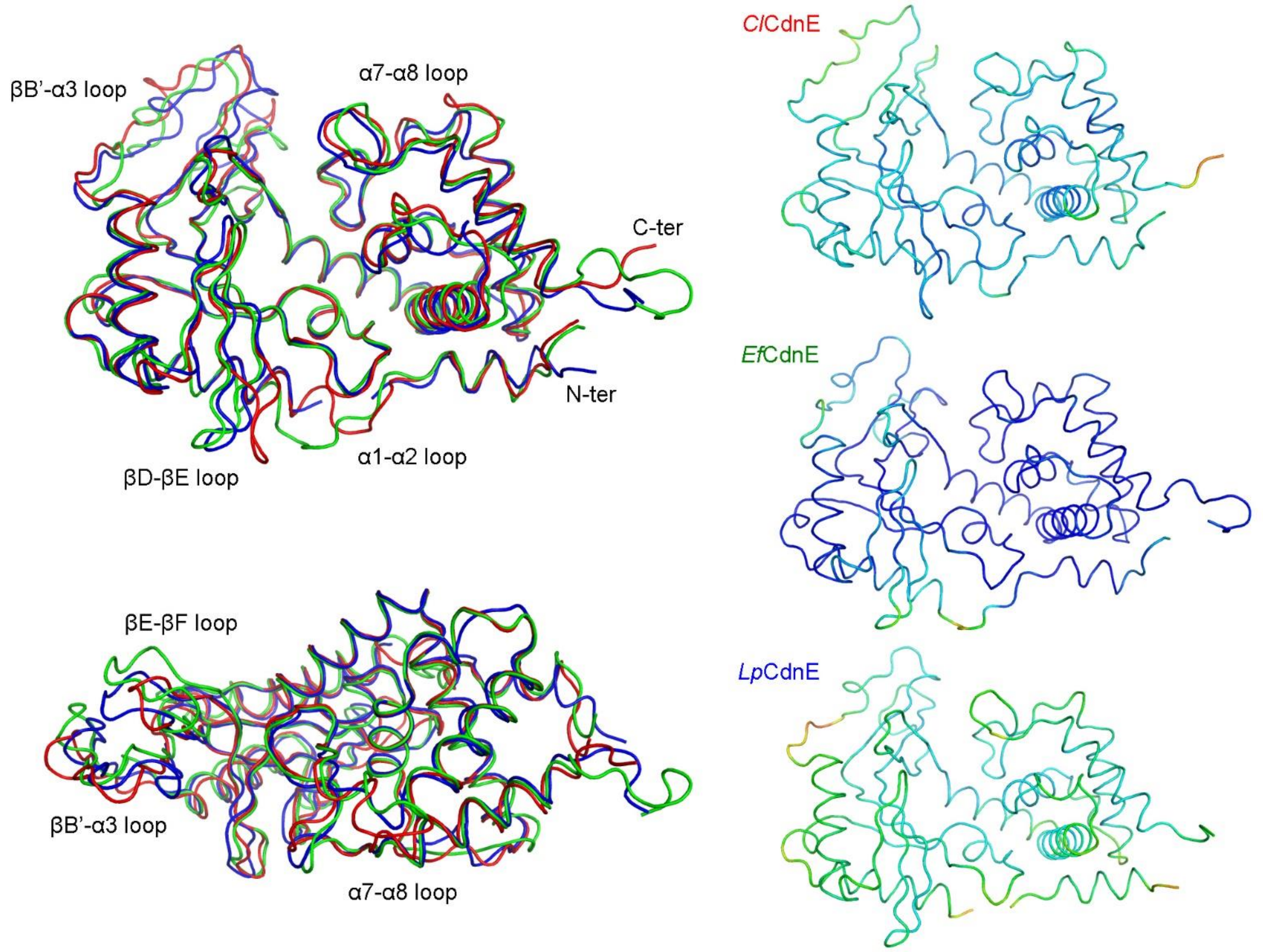


**Supplementary Figure 4. Structure comparison of *Cl*CdnE, *Ef*CdnE and *Lp*CdnE.**

The three protein chains are superimposed and shown as worm diagrams. On the left side are two roughly orthogonal views of the structures, with *Cl*CdnE colored red, *Ef*CdnE green, and *Lp*CdnE blue. On the right side the protein chains are spectrum-colored according to temperature factors, while red representing the highest and blue the lowest.

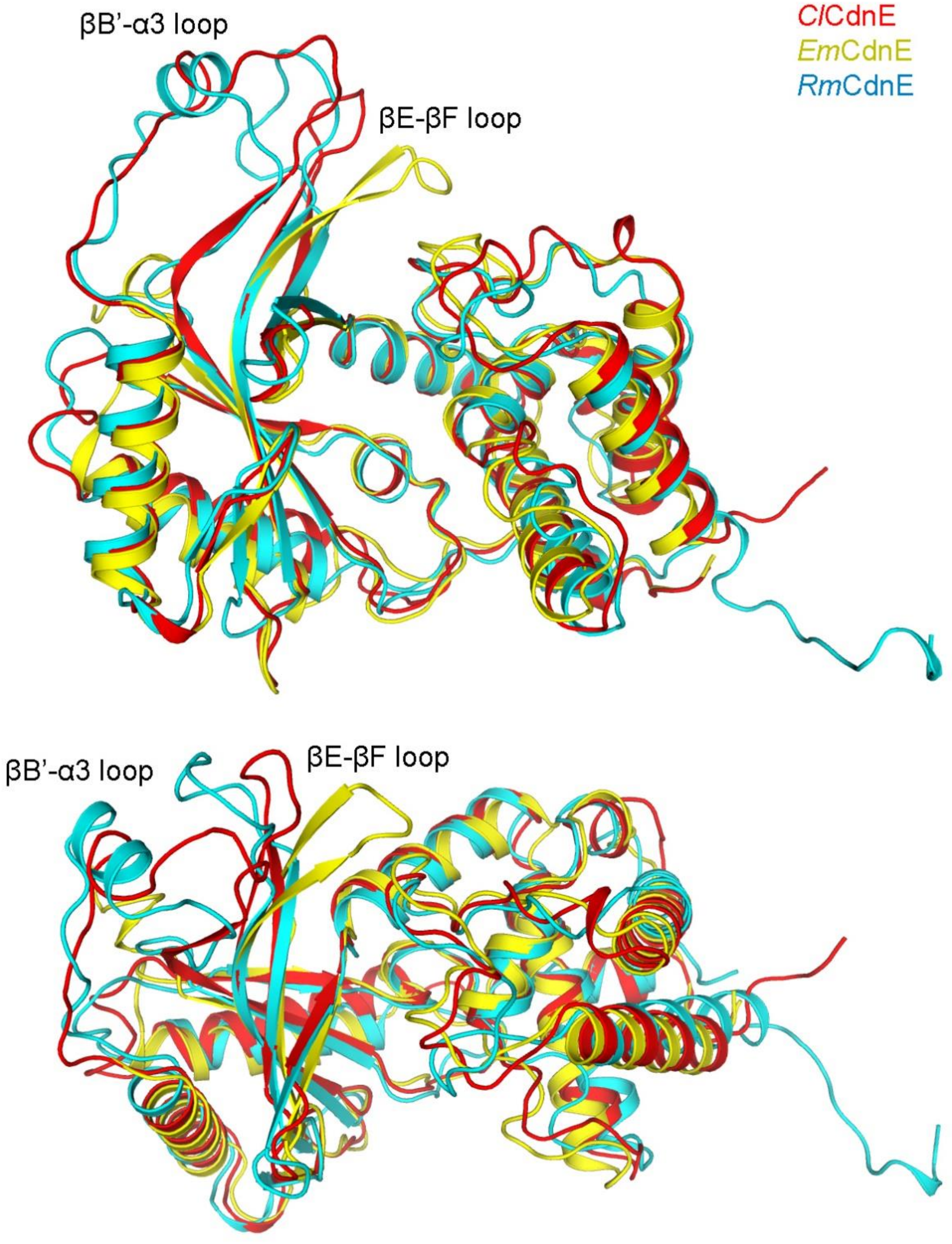


**Supplementary Figure 5. Structural comparison of *Cl*CdnE with *Em*CdnE and *Rm*CdnE (PDB 6E0M and 6E0K).**

The three native structures are superimposed and shown as ribbons diagrams in red, yellow and cyan and shown in two roughly orthogonal views.

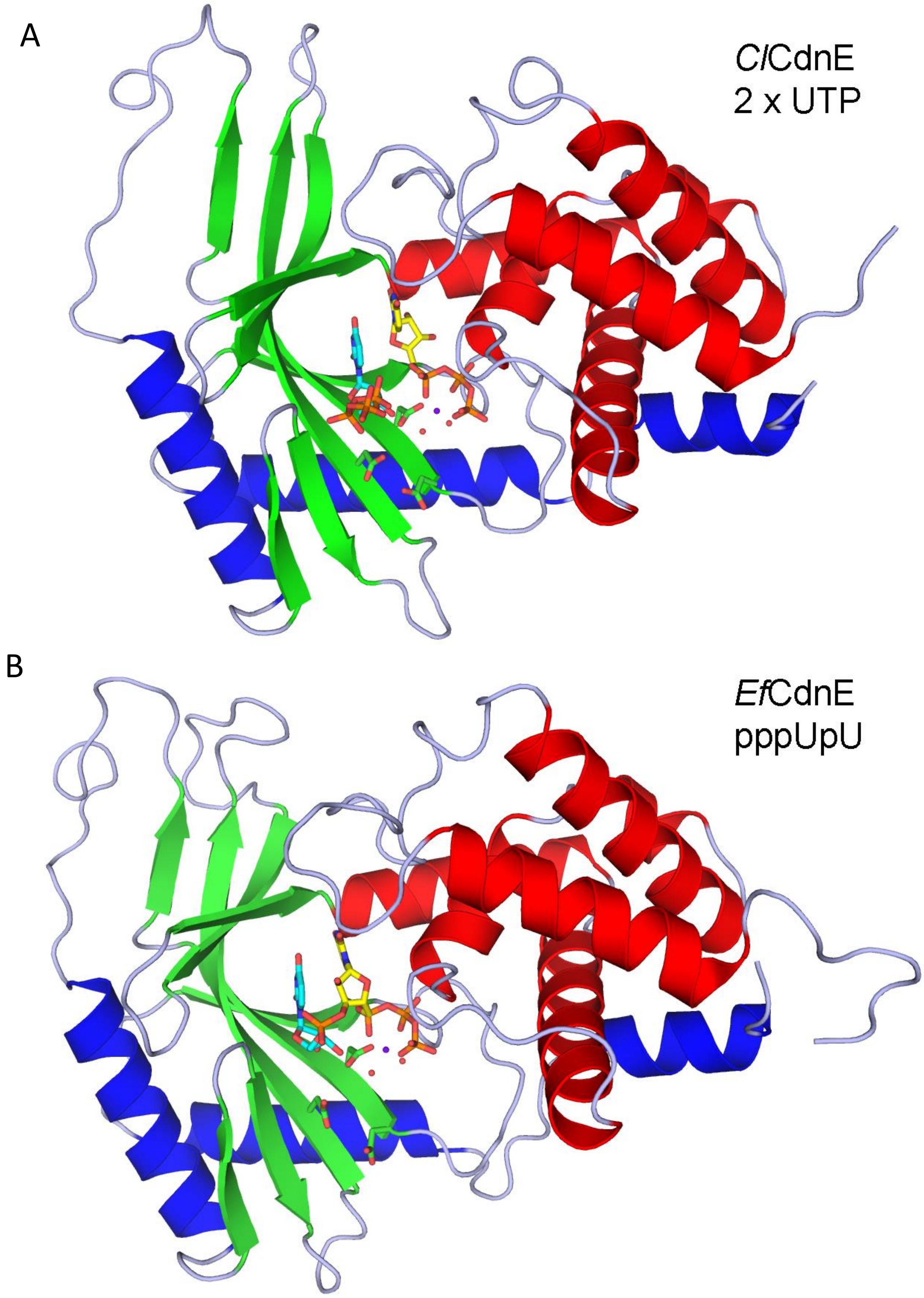


**Supplementary Figure 6. Structures of the (A) *Cl*CdnE-UTP and (B) *Ef*CdnE-pppUpU complexes.**
The proteins are shown as ribbons diagrams in a similar way as in Figure 1. The nucleotide ligands and the three catalytic aspartate side chains are shown as stick models and the Mg and waters as spheres. The carbon atoms in the donor and acceptor substrates in *Cl*CdnE are colored yellow and cyan, and so are the corresponding atoms in the reaction intermediate in *Ef*CdnE.

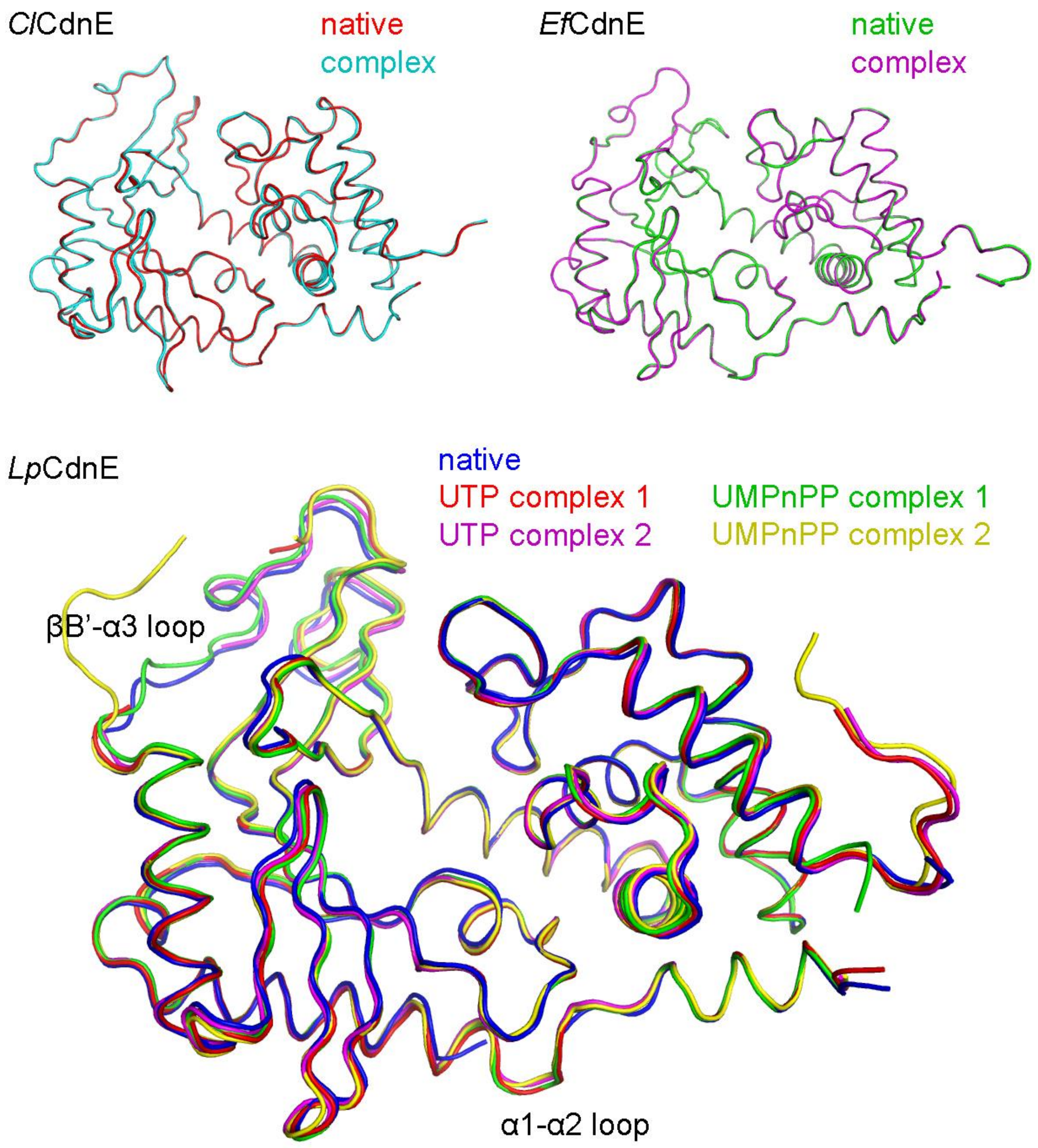


**Supplementary Figure 7. Comparison of the native and complex CdnE structures.**

In the upper panels the structures of *Cl*CdnE and *Ef*CdnE are superimposed separately and shown as worm diagrams in different colors. These structures are virtually identical, as expected from the crystal isomorphism. In the lower panel the *Lp*CdnE structures are shown. The native structure is disordered in the loop region between helices α1 and α2, whereas three of the four complex structures show disorder in the βB'-α3 loop. Structures in the C-terminal region also vary significantly.

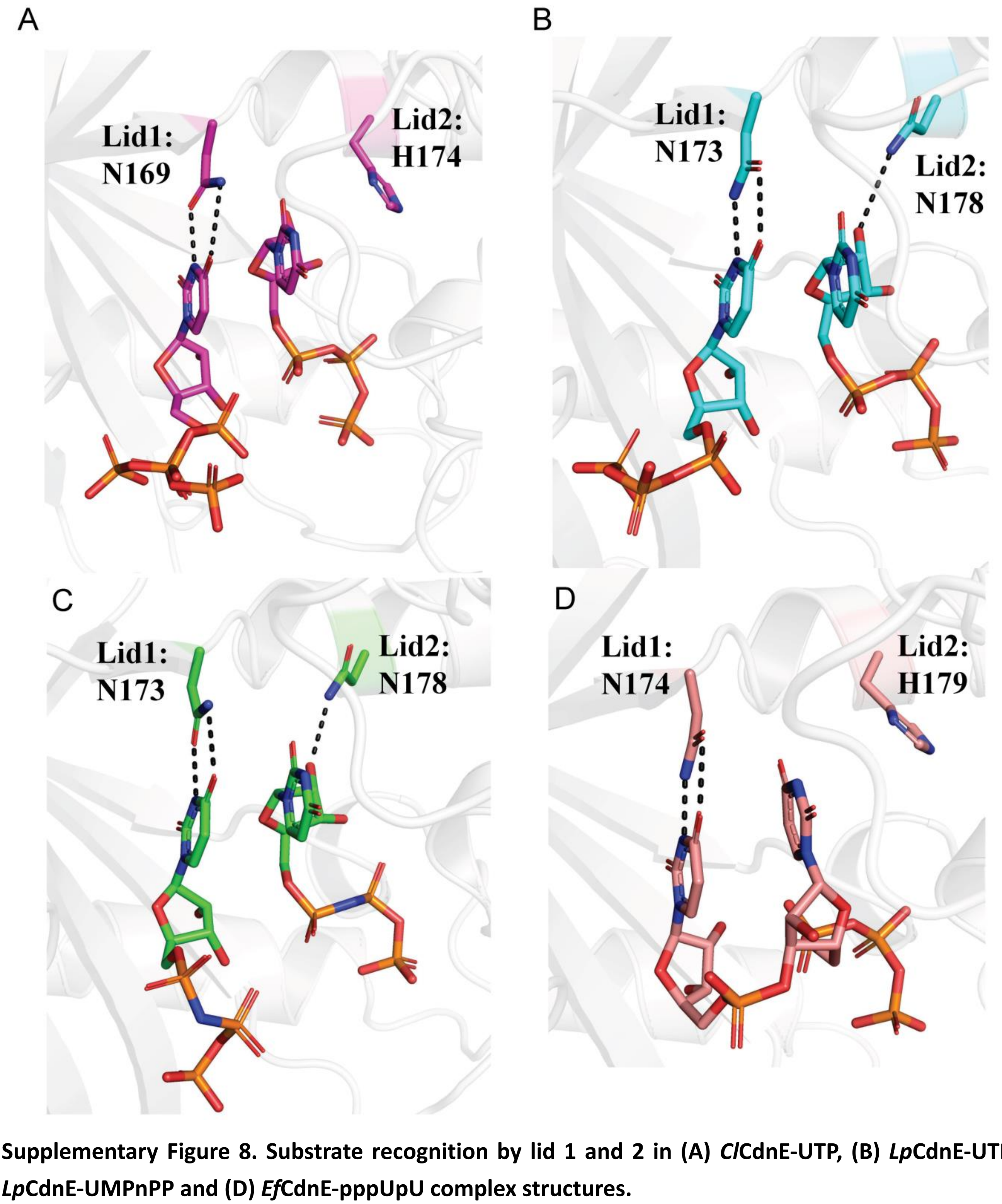


**Supplementary Figure 8. Substrate recognition by lid 1 and 2 in (A) *Cl*CdnE-UTP, (B) *Lp*CdnE-UTP, (C) *Lp*CdnE-UMPnPP and (D) *Ef*CdnE-pppUpU complex structures.**

The UTP, UMPnPP, pppUpU, lid 1 and lid 2 were shown in sticks. The hydrogen bonds were shown in black dashed lines.

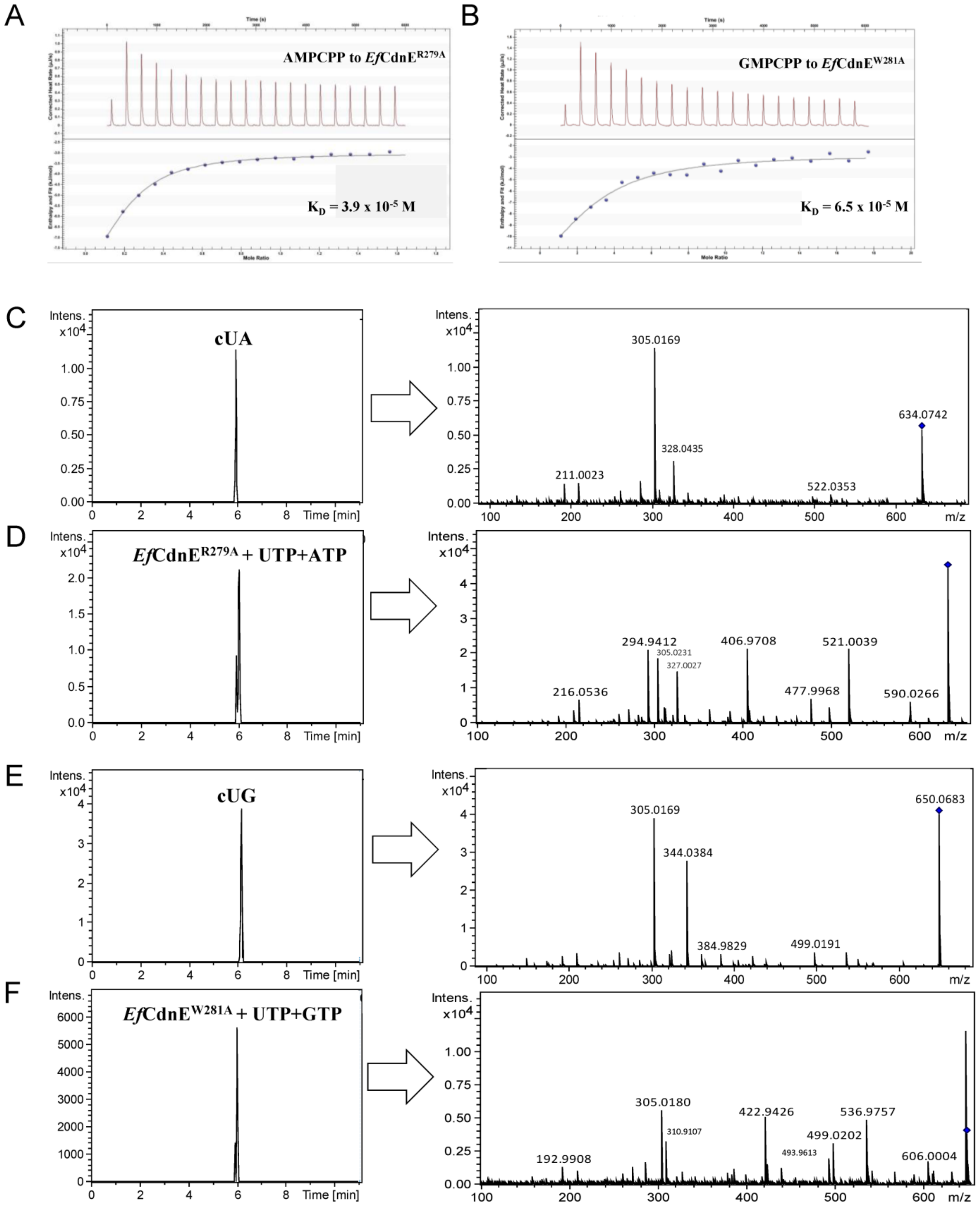


**Supplementary Figure 9. Functional validation of the critical role of (R/Q)xW motif of *Ef*CdnE.**
(A-B) ITC analysis of the interaction between (A) non-hydrolysable AMPcPP and *Ef*CdnE$^{R279A}$ and (B) non-hydrolysable GMPcPP and *Ef*CdnE$^{W281A}$. The determined dissociation constants ($K_D$, M) were indicated. (C) LC-MS/MS result of cyclic UMP-AMP (theoretical $[M-H]^-$: m/z 634.077), which is eluted at 6.1 min and has

fragmented ions of m/z 211.0023, m/z 305.0169, m/z 328.0435 and m/z 522.0353. (D) A peak of m/z 633.03 and its fragmented ions was found to be eluted at 6.1 min having the same elution time of cyclic UMP-AMP. (E) LC-MS/MS result of cyclic UMP-GMP (theoretical $[M-H]^-$: m/z 650.0643), which is eluted at 6.1 min and has fragmented ions of m/z 305.0169, m/z 344.0384, m/z 384.9829 and m/z 499.0191. (F), a peak of m/z 649.0 and its fragmented ions was found to be eluted at 6.1 min having the same elution time of cyclic UMP-GMP.

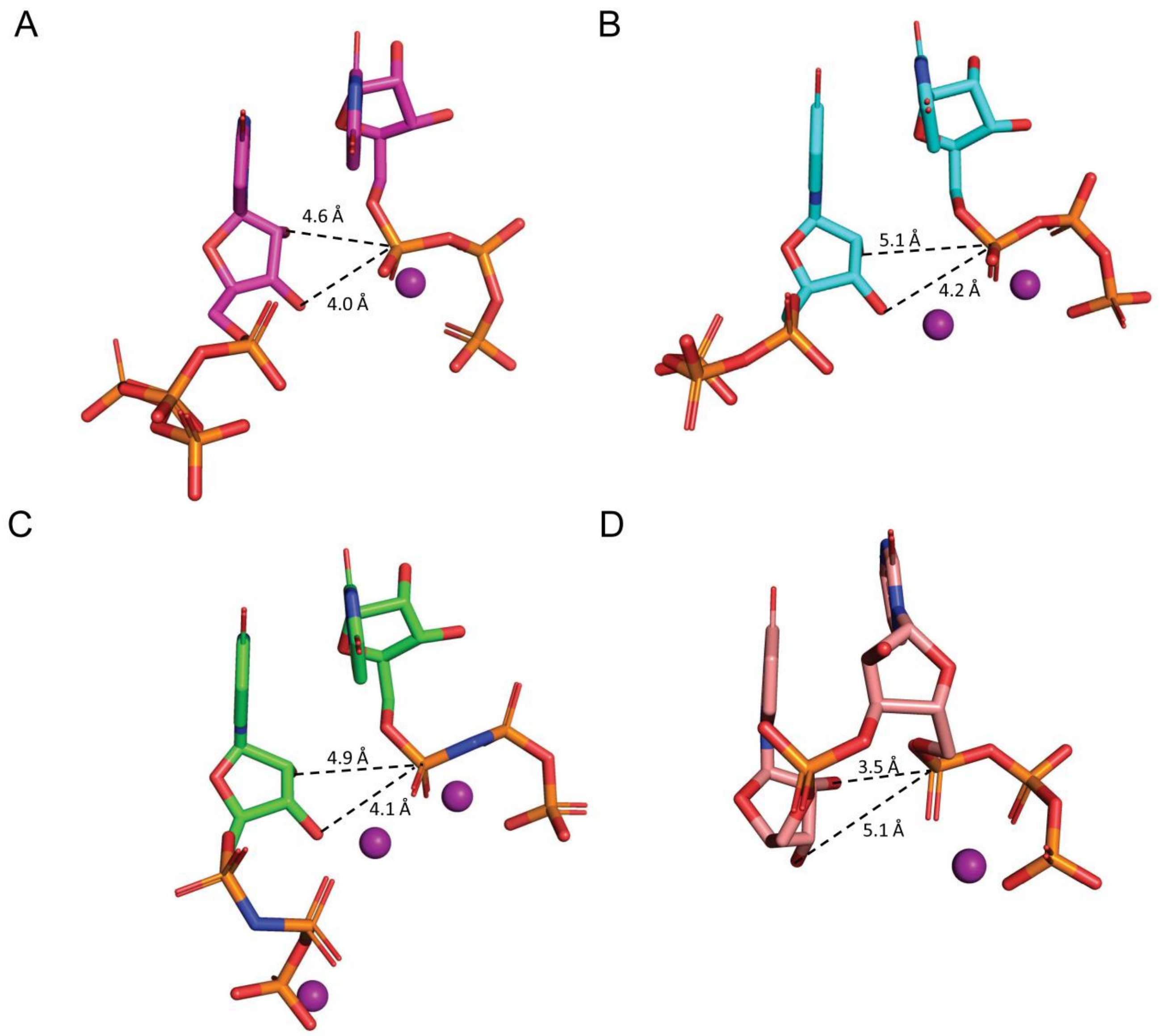


**Supplementary Figure 10. The enlarged view of the bound ligand in (A) *Cl*CdnE-UTP, (B) *Lp*CdnE-UTP, (C) *Lp*CdnE-UMPnPP and (D) *Ef*CdnE-pppUpU complex structures.**

The distances (Å) between 2'-OH/3'-OH of acceptor nucleotide and $\alpha$-phosphate of the donor nucleotide were shown. The UTP, UMPnPP and pppUpU were shown in sticks. The Mg ions were shown in purple spheres.

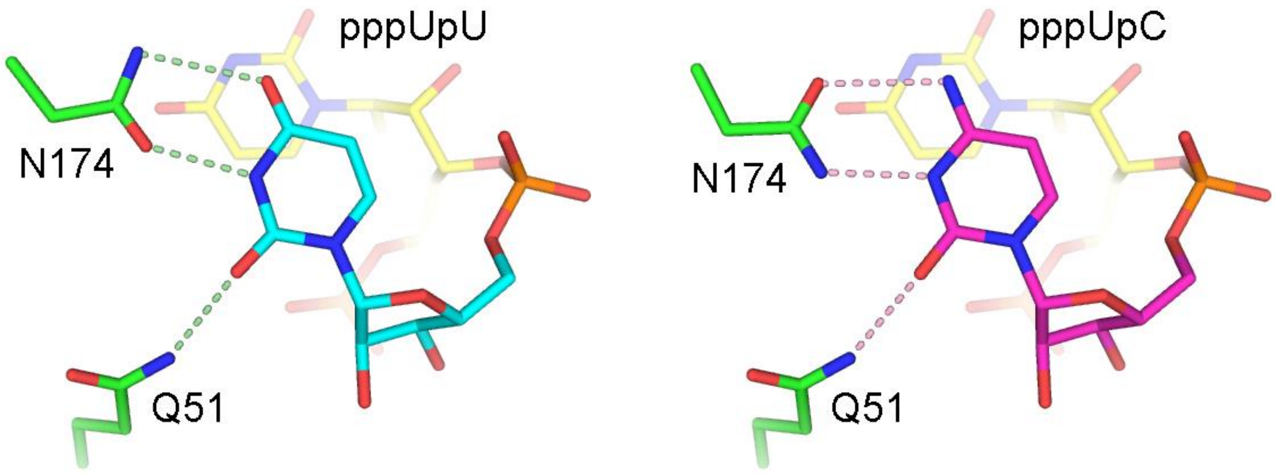


**Supplementary Figure 11. Acceptor pyrimidine binding modes of *Ef*CdnE.**

The left panel shows the hydrogen bonding interactions observed in the intermediate complex of *Ef*CdnE with pppUpU. In the right panel the acceptor nucleobase is replaced by a cytosine and the side chain of Asn174 is flipped over for proper hydrogen bond formations.

## Supplementary Tables

**Supplementary Table 1.** Data collection and refinement statistics from the *Cl*CdnE crystals.

| | **Native (PDB 7X4A)** | **UTP complex (PDB 7X4G)** |
|---|---|---|
| **Data collection** | | |
| Space group | I23 | I23 |
| Cell dimensions | | |
| *a*, *b*, *c* (Å) | 132.1, 132.1, 132.1 | 132.6, 132.6, 132.6 |
| α, β, γ (°) | 90, 90, 90 | 90, 90, 90 |
| Resolution (Å) | 30-2.20 (2.28-2.20)* | 30-2.60 (2.69-2.60) |
| Unique reflections | 19381 (1920) | 12099 (1180) |
| Completeness (%) | 100.0 (100.0) | 100.0 (100.0) |
| Redundancy | 21.2 (17.7) | 22.5 (22.6) |
| $R_{merge}$ (%) | 8.7 (61.7) | 8.1 (187.4) |
| $R_{pim}$ (%) | 1.9 (15.0) | 1.7 (40.3) |
| I/σ *(I)* | 38.9 (5.5) | 50.1 (2.1) |
| $CC_{1/2}$ | 0.988 (0.951) | 0.950 (0.729) |
| **Refinement** | | |
| No. reflections | 19332 (1914) | 12041 (1126) |
| $R_{work}$ / $R_{free}$ | 0.157 / 0.215 | 0.168 / 0.201 |
| No. atoms | | |
| Protein | 2472 | 2472 |
| Ligand/ion | - | 59 |
| Water | 295 | 221 |
| *B*-factors | | |
| Protein | 35.3 | 53.5 |
| Ligand/ion | - | 52.8 |
| Water | 44.8 | 49.1 |
| R.m.s. deviations | | |
| Bond lengths (Å) | 0.0092 | 0.0018 |
| Bond angles (°) | 0.97 | 0.50 |

*Values in parentheses are for highest-resolution shell.

**Supplementary Table 2**. Data collection and refinement statistics from the *Ef*CdnE crystals.

| | **Native (PDB 7X4C)** | **pppU[3’-5’]pU complex (PDB 7X4P)** | **pppU[2’-5’]p complex (PDB 8HYK)** |
|---|---|---|---|
| **Data collection** | | | |
| Space group | $P2_1$ | $P2_1$ | $P2_1$ |
| Cell dimensions | | | |
| *a*, *b*, *c* (Å) | 41.3, 57.1, 64.8 | 41.3, 56.8, 64.8 | 41.4, 56.9, 64.8 |
| α, β, γ (°) | 90, 97.1, 90 | 90, 97.2, 90 | 90, 97.0, 90 |
| Resolution (Å) | 30-1.75 (1.81-1.75) | 30-1.60 (1.66-1.60) | 30-2.02 (2.09-2.02) |
| Unique reflections | 29511 (2981) | 38096 (3716) | 19291 (1849) |
| Completeness (%) | 97.2 (98.0) | 96.6 (95.0) | 97.8 (94.8) |
| Redundancy | 3.4 (3.3) | 3.9 (3.9) | 5.9 (6.2) |
| $R_{merge}$ (%) | 3.3 (7.5) | 3.5 (6.8) | 6.7 (16.2) |
| $R_{pim}$ (%) | 2.2 (4.9) | 2.1 (4.0) | 3.0 (7.0) |
| I/σ *(I)* | 35.1 (15.1) | 33.1 (18.9) | 24.5 (10.4) |
| $CC_{1/2}$ | 0.995 (0.988) | 0.996 (0.993) | 1.000 (0.982) |
| **Refinement** | | | |
| No. reflections | 29445 (2943) | 38076 (3676) | 19273 (1840) |
| $R_{work}$ / $R_{free}$ | 16.8 / 19.8 | 14.9 / 18.8 | 17.5 / 18.6 |
| No. atoms | | | |
| Protein | 2525 | 2545 | 2513 |
| Ligand/ion | 10 | 50 | 34 |
| Water | 418 | 509 | 187 |
| *B*-factors | | | |
| Protein | 20.6 | 16.5 | 28.46 |
| Ligand/ion | 23.5 | 27.8 | 29.52 |
| Water | 30.8 | 30.4 | 30.30 |
| R.m.s. deviations | | | |
| Bond lengths (Å) | 0.0043 | 0.0093 | 0.015 |
| Bond angles (°) | 0.65 | 1.06 | 1.71 |

*Values in parentheses are for highest-resolution shell.

**Supplementary Table 3.** Data collection and refinement statistics from the *Lp*CdnE crystals.

| | **Native (PDB 7X4F)** | **UTP complex (PDB 7X4Q)** | **UMPNPP complex (PDB 7X4T)** |
|---|---|---|---|
| **Data collection** | | | |
| Space group | $P3_1$ | $P2_1$ | $P2_12_12$ |
| Cell dimensions | | | |
| *a*, *b*, *c* (Å) | 60.5, 60.5, 100.6 | 65.6, 74.7, 80.3 | 72.9, 157.5, 66.3 |
| α, β, γ (°) | 90, 90, 120 | 90, 98.2, 90 | 90, 90, 90 |
| Resolution (Å) | 30-2.45 (2.54-2.45) | 30-1.95 (2.02-1.95) | 30-2.20 (2.28-2.20) |
| Unique reflections | 14892 (1507) | 56124 (5577) | 40378 (3848) |
| Completeness (%) | 99.0 (100.0) | 100.0 (100.0) | 98.9 (99.2) |
| Redundancy | 3.7 (3.8) | 3.8 (3.8) | 6.1 (5.8) |
| $R_{merge}$ (%) | 7.7 (62.9) | 7.5 (51.3) | 5.5 (21.3) |
| $R_{pim}$ (%) | 4.7 (37.8) | 4.4 (30.6) | 2.3 (9.3) |
| $I/\sigma(I)$ | 16.5 (2.1) | 17.5 (2.5) | 29.3 (8.9) |
| $CC_{1/2}$ | 0.927 (0.652) | 0.956 (0.857) | 0.992 (0.979) |
| **Refinement** | | | |
| No. reflections | 14466 (1269) | 52948 (3930) | 39127 (3731) |
| $R_{work}$ / $R_{free}$ | 19.1 / 22.3 | 16.1 / 18.7 | 15.9 / 19.7 |
| No. atoms | | | |
| Protein | 2362 | 4748 | 4806 |
| Ligand/ion | 35 | 127 | 143 |
| Water | 197 | 591 | 543 |
| *B*-factors | | | |
| Protein | 43.8 | 24.6 | 36.9 |
| Ligand/ion | 91.5 | 20.0 | 37.1 |
| Water | 47.5 | 35.5 | 41.8 |
| R.m.s. deviations | | | |
| Bond lengths (Å) | 0.0025 | 0.0072 | 0.0043 |
| Bond angles (°) | 0.48 | 0.88 | 0.70 |

*Values in parentheses are for highest-resolution shell.

**Supplementary Table 4.** Cα deviation and sequence identity. The structures of native proteins are compared and the resulting rmsd between matched Cα pairs and sequence identity are listed.

| | *Cl*CdnE | *Ef*CdnE | *Lp*CdnE |
|---|---|---|---|
| *Em*CdnE | 1.50 Å/256 Cα; 33% ID | 1.83 Å/255 Cα; 33% ID | 1.64 Å/244 Cα; 32% ID |
| *Rm*CdnE | 1.74 Å/263 Cα; 25% ID | 1.63 Å/271 Cα; 27% ID | 1.75 Å/269 Cα; 31% ID |
| *Cl*CdnE | | 1.38 Å/280 Cα; 44% ID | 1.32 Å/276 Cα; 36% ID |
| *Ef*CdnE | | | 1.45 Å/278 Cα; 34% ID |

**Supplementary Table 5.** Cα deviation between native and complex *Lp*CdnE structures. Numbers in the diagonal boxes are for the residues in the proteins. The rmsd (in Å) between different protein molecules are listed in the upper triangle, and the corresponding numbers of matched Cα pairs are listed in the lower triangle.

| | native | UTP (A) | UTP (B) | UMPnPP (A) | UMPnPP (B) |
|---|---|---|---|---|---|
| native | 292 | 0.732 | 0.563 | 0.824 | 0.685 |
| UTP (A) | 283 | 291 | 0.396 | 0.515 | 0.429 |
| UTP (B) | 286 | 289 | 294 | 0.500 | 0.404 |
| UMPnPP (A) | 281 | 290 | 288 | 298 | 0.458 |
| UMPnPP (B) | 286 | 283 | 286 | 281 | 292 |

**Supplementary Table 6.** Distances (Å) between the bound Mg ions and the coordinating atoms in various CdnE complex structures. The representative residue numbers of the interacting amino acid residues are from *Lp*CdnE.

| | *Cl*-UTP | *Ef*-pppUpU | *Lp*-UTP 1 | *Lp*-UTP 2 | *Lp*-UMPnPP 1 | *Lp*-UMPnPP 2 |
|---|---|---|---|---|---|---|
| Mg-A | | | | | | |
| α-phosphate[d] | | | 2.47 | 2.48 | 2.44 | 2.52 |
| Asp67 | | | 2.34 | 2.31 | 2.35 | 2.35 |
| Asp139 | | | 2.38 | 2.38 | 2.35 | 2.41 |
| ribose O3’[a] | | | 2.36 | 2.45 | 2.20 | 2.17 |
| water #2 | | | 2.53 | 2.43 | 2.45 | 2.43 |
| water #3 | | | 2.40 | 2.42 | 2.53 | 2.55 |
| Mg-B | | | | | | |
| α-phosphate[d] | 2.06 | 2.09 | 2.09 | 2.05 | 2.00 | 2.03 |
| β-phosphate[d] | 2.13 | 2.01 | 2.06 | 2.06 | 2.06 | 2.14 |
| γ-phosphate[d] | 1.97 | 1.94 | 2.02 | 2.03 | 2.11 | 1.98 |
| Asp67 | 1.97 | 2.06 | 2.11 | 2.08 | 2.04 | 2.04 |
| water #1 | 2.10 | 2.12 | 2.03 | 2.24 | 2.15 | 2.14 |
| water #2 | 2.06 | 2.08 | 2.12 | 2.09 | 2.16 | 2.23 |
| Mg-C | | | | | | |
| γ-phosphate[a] | | | | | 2.34 | |
| Asp65 | | | | | 2.16 | |
| Asp128 | | | | | 2.20 | |
| Asp139 | | | | | 2.24 | |
| water #4 | | | | | 2.07 | |
| water #5 | | | | | 2.20 | |

[a] from the acceptor nucleotide; [d] from the donor nucleotide.

**Supplementary Table 7.** The sequences of the codon-optimized genes used for construction into vector pET21 in this study.

| |
|---|
| ***Lp*CdnE** |
| CAT ATG AGC ATT GAT TGG GAA CAG ACC TTT CGC AAA TGG AGC AAA CCG AGC AGC GAA ACC GAA AGC ACC AAA GCG GAA AAC GCG GAA CGC ATG ATT AAA GCG GCG ATT AAC AGC AGC CAG ATT CTG AGC ACC AAA GAT ATT AGC GTG TTT CCG CAG GGC AGC TAT CGC AAC AAC ACC AAC GTG CGC GAA GAT AGC GAT GTG GAT ATT TGC GTG TGC CTG AAC ACC CTG GTG CTG AGC GAT TAT AGC CTG GTG CCG GGC ATG AAC GAT AAA CTG GCG GAA CTG CGC ACC GCG AGC TAT ACC TAT AAA CAG TTT AAA AGC GAC CTG GAG ACT GCG CTG AAA AAC AAA TTT GGC ACC CTG GGC GTG AGC CGC GGC GAT AAA GCG TTT GAT GTG CAT GCG AAC AGC TAT CGC GTG GAT GCG GAT GTG GTG CCG GCG ATT CAA GGT CGT TTA TAT TAT GAT AAA AAC CAC AAC GCG TTC ATC CGC GGC ACC TGC ATT AAA CCG GAT AGC GGC GGT ACC ATT TAT AAC TGG CCG GAA CAG AAC TAT AGC AAC GGC GTG AAC AAA AAC AAA AGC ACC GGC AAC CGC TTT AAA CTG ATT GTG CGC GCG ATT AAA CGC CTG CGC AAC CAT CTG GCG GAA AAA GGC TAT AAC ACC GCG AAA CCG ATT CCG AGC TAT CTG ATG GAA TGC CTG GTG TAT ATT GTG CCG GAT CAG TAT TTT ACC GGC GAT AGC TAT AAA ACC AAC GTG GAA AAC TGC ATT AAC TAC CTG TAT AAC CAG ATC GAT AGC AGC GAT TGG ACC GAA ATT AAC GAA ATT AAA TAC CTG TTC GGC AGC CAC CAG ATG TGG AAC AAA ACC CAG GTG AAA GAA TTT CTG CTG ACC GCG TGG AGC TAT ATT CAG AAA AAC CTC GAG |
| ***Cl*CdnE** |
| CAT ATG GCG AAA TAT ACC GAA GAT CAG CTG ACC AGC TGG ACC AAA CCG CCG AGC GAT AGC GAA CAG ACC AAA CTG GAA AAC AGC GAA AAA ATG GTG CGC GAA GCG ATT AGC AGC GAT GAA AAA CTG AGC AAA AAA ACC ATT GAG ACT TTC GGC CAG GGC AGC TAT GCG AAC AAC ACC AAC GTG CGC CTG AAC AGC GAT ATT GAT ATT AAC GTG AAA TAC AGC GAC GGC TTC TAT TTC GAC CTG CCG AAA GAT AAA AGC CGC GAA GAT TTT GGC ATT ACC CTG ACC AGC TAT AGC TAT GAA GAA TAT AAA GAC GAC GTG GAG AAC GCG CTG GTG AAC AAA TTT GGC CGC AGC GAA GTG GTG CGC AAA GAT AAA TGC ATT ACC GTG AAA GAA AAC AGC TAC CGC GTG GAA ACC GAT GTG GTG CCG ACC TGG GAT TAT CGC CGC TAT AGC GAA AAC GGC AAC TAT GTG CAG GGC ACC AAA TTT AAA ACC GAT AAA GGC ATT TGG ATC GAC AAC TAC CCG AAA CAG CAT ATT GCG AAC GGC ATT AGC AAA AAC AAC AAC ACC GCG CGC CGC TTT AAA CGC CTG ACC CGT TTA CAT CGC AAA CTG CGC TAT AAA ATG ATT GAC GAC GGC GGC AAC GTG AGC GAT AAC ATT ACC AGC TTT CTG CTG GAA TGC CTG GTG TGG AAC GTG CCG AAT CGC ATT ATG AAC GAT TAT GAT ACC TGG ACC GAA CGC CTG AAA CAG AGC ATT ATT TAT CTG TAT AAC AAC ACC CGC GAG GAG AGC AGC TGC AAA GAA TGG GGC GAA GTG AGC GAA CTG CTG TAT CTG TTT CAT GGC GGC CGC AAA TGG ACC AGC AAA GAT GTG AAC AGC TAT ATG GTG CTG CTG TGG AAC CAT CTG GAA TTT CTC GAG |
| ***Ef*CdnE** |
| CAT ATG AGC AAA TTT AGC GAA AGC ACC CTG AGC GGC TGG ACC AAA CCG GCG AGC GTT ACC GAA GAA GAT CGC ATT GAA AAC ACC ATT AGC ATG ATT AAG AGC GCG ATT AAG AAC GAC AAC AAC TTT GAC AAC CTG GTG TAT GAA GTG TTC GTG CAG GGC AGC TAT GGC AAC AAC ACC AAC GTG CGC ACC |

| |
|---|
| AAC AGC GAT ATT GAT GTG AAC ATT ATG CTG ACC AGC ACC TTT TAT AGC AAA TAC CCG GAA GGC<br>AAA ACC AAC AGC GAT TAT GGC TTT ACC GAT GGC ACC ATT ACC TAT AAC GAA TAT AAA AAC CTG<br>ATC CTG ACC GCG CTG ACC AAC AAA TTT GGC ACC GGC AAC GTG ACC GTG GGC AAC AAA AGC ATT<br>AAA ATT ACC AGC AAC AGC TAC CGC GTG GAA GCG GAT TGC ATT CCG AGC CTG TTA TAT CGC AAC<br>TAT GAA TAT GAA AAC AGC AGC AGC CCG AAC AAC TAT ATT GAA GGC ATT AAA TAC TTC GCG AGC<br>GAC AAC ACC AGC GTG GTG AAC TAT CCG AAA GTG CAT ATT AAC AAC GGC ATT GAG AAA AAC AAC<br>CAG ACC CAT AAA AAC TAC AAG CGC CTG GTG CGC GTG ATT AAA CGC CTG CGC AAT AAA ATG ACC<br>GCG GAA AAC CAT TTT ACC AAC GAA AAC ATT ACC AGC TTC CTG ATT GAA TGC CTG ATT TGG AAC<br>GTG CCG AAC AAC TAT ATT AAC GAC TAT GAC ACC TGG GAC GAG ACT ATT AAA CAG ACC CTG ATT<br>TTT ATT AAG AGC AGC ATC AAC GAT AAC AGC TAC AAG AAC TGG ACC GAA GTG AGC GGC ATG TTT<br>TAT CTG TTT CAT AAC AAC CGC AAA TGG ACC AGC GAT GAT GTG AGC AGC TTT GTG AAC AGC CTG<br>TGG AGC TTT ATG GAA TAT CTC GAG |
| ***Ef*CdnE$^{R279A}$** |
| CAT ATG AGC AAA TTT AGC GAA AGC ACC CTG AGC GGC TGG ACC AAA CCG GCG AGC GTT ACC GAA<br>GAA GAT CGC ATT GAA AAC ACC ATT AGC ATG ATT AAG AGC GCG ATT AAG AAC GAC AAC AAC TTT<br>GAC AAC CTG GTG TAT GAA GTG TTC GTG CAG GGC AGC TAT GGC AAC AAC ACC AAC GTG CGC ACC<br>AAC AGC GAT ATT GAT GTG AAC ATT ATG CTG ACC AGC ACC TTT TAT AGC AAA TAC CCG GAA GGC<br>AAA ACC AAC AGC GAT TAT GGC TTT ACC GAT GGC ACC ATT ACC TAT AAC GAA TAT AAA AAC CTG<br>ATC CTG ACC GCG CTG ACC AAC AAA TTT GGC ACC GGC AAC GTG ACC GTG GGC AAC AAA AGC ATT<br>AAA ATT ACC AGC AAC AGC TAC CGC GTG GAA GCG GAT TGC ATT CCG AGC CTG TTA TAT CGC AAC<br>TAT GAA TAT GAA AAC AGC AGC AGC CCG AAC AAC TAT ATT GAA GGC ATT AAA TAC TTC GCG AGC<br>GAC AAC ACC AGC GTG GTG AAC TAT CCG AAA GTG CAT ATT AAC AAC GGC ATT GAG AAA AAC AAC<br>CAG ACC CAT AAA AAC TAC AAG CGC CTG GTG CGC GTG ATT AAA CGC CTG CGC AAT AAA ATG ACC<br>GCG GAA AAC CAT TTT ACC AAC GAA AAC ATT ACC AGC TTC CTG ATT GAA TGC CTG ATT TGG AAC<br>GTG CCG AAC AAC TAT ATT AAC GAC TAT GAC ACC TGG GAC GAG ACT ATT AAA CAG ACC CTG ATT<br>TTT ATT AAG AGC AGC ATC AAC GAT AAC AGC TAC AAG AAC TGG ACC GAA GTG AGC GGC ATG TTT<br>TAT CTG TTT CAT AAC AAC GCC AAA TGG ACC AGC GAT GAT GTG AGC AGC TTT GTG AAC AGC CTG<br>TGG AGC TTT ATG GAA TAT CTC GAG |
| ***Ef*CdnE$^{W281A}$** |
| CAT ATG AGC AAA TTT AGC GAA AGC ACC CTG AGC GGC TGG ACC AAA CCG GCG AGC GTT ACC GAA<br>GAA GAT CGC ATT GAA AAC ACC ATT AGC ATG ATT AAG AGC GCG ATT AAG AAC GAC AAC AAC TTT<br>GAC AAC CTG GTG TAT GAA GTG TTC GTG CAG GGC AGC TAT GGC AAC AAC ACC AAC GTG CGC ACC<br>AAC AGC GAT ATT GAT GTG AAC ATT ATG CTG ACC AGC ACC TTT TAT AGC AAA TAC CCG GAA GGC<br>AAA ACC AAC AGC GAT TAT GGC TTT ACC GAT GGC ACC ATT ACC TAT AAC GAA TAT AAA AAC CTG<br>ATC CTG ACC GCG CTG ACC AAC AAA TTT GGC ACC GGC AAC GTG ACC GTG GGC AAC AAA AGC ATT<br>AAA ATT ACC AGC AAC AGC TAC CGC GTG GAA GCG GAT TGC ATT CCG AGC CTG TTA TAT CGC AAC<br>TAT GAA TAT GAA AAC AGC AGC AGC CCG AAC AAC TAT ATT GAA GGC ATT AAA TAC TTC GCG AGC<br>GAC AAC ACC AGC GTG GTG AAC TAT CCG AAA GTG CAT ATT AAC AAC GGC ATT GAG AAA AAC AAC<br>CAG ACC CAT AAA AAC TAC AAG CGC CTG GTG CGC GTG ATT AAA CGC CTG CGC AAT AAA ATG ACC |

GCG GAA AAC CAT TTT ACC AAC GAA AAC ATT ACC AGC TTC CTG ATT GAA TGC CTG ATT TGG AAC GTG CCG AAC AAC TAT ATT AAC GAC TAT GAC ACC TGG GAC GAG ACT ATT AAA CAG ACC CTG ATT TTT ATT AAG AGC AGC ATC AAC GAT AAC AGC TAC AAG AAC TGG ACC GAA GTG AGC GGC ATG TTT TAT CTG TTT CAT AAC AAC CGC AAA GCG ACC AGC GAT GAT GTG AGC AGC TTT GTG AAC AGC CTG TGG AGC TTT ATG GAA TAT CTC GAG

The sequences underline in red corresponds to the recognition site for *Nde*I.

The sequences underline in blue corresponds to the recognition site for *Xho*I.
